# Sex hormones and serotonin 4 receptor brain binding in men with and without major depressive disorder

**DOI:** 10.1016/j.nsa.2025.105517

**Published:** 2025-02-28

**Authors:** Malene Ravn Aarestrup, Kristian H. Reveles Jensen, Søren Vinther Larsen, Brice Ozenne, Kristin Köhler-Forsberg, Gitte Moos Knudsen, Martin Balslev Jørgensen, Vibe G. Frokjaer

**Affiliations:** a Neurobiology Research Unit, Rigshospitalet, Copenhagen, Denmark; b Department of Clinical Medicine, Faculty of Health and Medical Sciences, University of Copenhagen, Copenhagen, Denmark; c Department of Psychiatry, Psychiatric Centre Copenhagen, Copenhagen, Denmark; d Department of Public Health, Section of Biostatistics, University of Copenhagen, Copenhagen, Denmark

**Keywords:** 5-HT_4_ receptor, Positron emission tomography (PET), Major depressive Disorder (MDD), Estrogen, Testosterone, Sex Hormones

## Abstract

Sex hormones may contribute to the pathophysiology of Major Depressive Disorder (MDD) through effects on the serotonergic brain system. Estradiol is associated with serotonergic neurobiology in rodent models and humans across sexes. In healthy men, we have previously observed a negative association between testosterone and serotonin 4 receptor (5-HT4R) levels, a receptor implicated in depression and a promising novel antidepressant target. Here, we investigate the associations between sex hormone levels and 5-HT4R binding in unmedicated men with MDD compared to healthy men.

We used a latent variable model to evaluate the association between estradiol and testosterone, and cerebral 5-HT4R binding based on [^11^C]SB207145 Positron Emission Tomography (PET) data from 25 males with MDD from the Neuropharm trial and 52 healthy males (aged 18–49), which included 38 healthy males from our previous study.

As previously shown in the complete group of men and women with MDD from the Neuropharm trial, we here confirm that a lower cerebral 5-HT4R level is also observed in the male MDD population alone compared to healthy men (β = −0.07, *p* = 0.012). In addition to the previously shown negative association between global 5-HT4R levels and testosterone (β = −0.007, *p* = 0.006) in healthy men, we also observe a positive association with estradiol (β = 1.74, *p* = 0.001). However, we do not observe this in men with MDD (testosterone: β = 0.0001, *p* = 0.97; estradiol: β = 0.64, *p=*0.36). There was a trend towards a group difference in the association between testosterone and global 5-HT4R (β = 0.008, *p* = 0.082).

In summary, we found evidence for a coupling between 5-HT4R and sex hormone levels in healthy men. This neuroendocrine connection appeared unclear in unmedicated men with depression, possibly due to a different and weaker coupling.

## Highlights

• Men with MDD have lower cerebral 5-HT_4_ receptor binding compared to healthy men.
• Estradiol and testosterone were associated with cerebral 5-HT_4_ receptor binding in healthy men.
• In contrast, we observe some evidence for an uncoupling between testosterone and 5-HT4_4_ receptor binding in unmedicated men with depression.

## 1 Introduction

Sex hormone transitions are suggested to play a role in depressive episodes in women (Frokjaer, 2020); the role of sex hormones in depression in men is, however, sparsely studied (Rosa and Bücker, 2024; Thériault and Perreault, 2019; Brietzke et al., 2019). In men above 70 years of age, low testosterone has been associated with Major Depressive Disorder (MDD) (Almeida et al., 2008). Also, testosterone replacement therapy has been shown to reduce depressive symptoms in 18 to 70-year-old men (Zarrouf et al., 2009). Men and postmenopausal women respond poorer to Selective Serotonin Reuptake Inhibitors (SSRI) than women in their reproductive age (LeGates et al., 2018). Further, middle-aged men with MDD treated with an SSRI appear to have lower plasma estradiol levels relative to healthy men and with plasma estradiol negatively correlated with their depression severity (Arinami et al., 2021).

Sex hormone receptors are abundant in brain regions considered important for mood regulation, such as the hippocampus and amygdala (McHenry et al., 2014), and are expressed by serotonergic neurons (Barth et al., 2015). Thus, sex hormones can play a role in mood regulation through modulation of the serotonergic neurotransmission (Cahill, 2006; Cosgrove et al., 2007).

The postsynaptic serotonin 4 receptor (5-HT4R) has been implicated in familial risk for depression (Madsen et al., 2015) and depression pathology (Rebholz et al., 2018; Köhler-Forsberg et al., 2023). 5-HT4R density is particularly high in the striatal and limbic areas, which are involved in mood, hedonic health, and cognition (Murphy et al., 2021), and it is a promising treatment target for MDD (Mendez-David et al., 2014a; Segi-Nishida, 2017; Frokjaer, 2023).

Three weeks of 10.13039/100017455SSRI intake in healthy individuals reduced brain 5-HT4R levels, presumably due to increased 5-10.13039/100014246HT brain tonus (Haahr et al., 2014) and, consequently, a 5-HT4R downregulation, an interpretation supported by rodent studies (Jennings et al., 2012; Licht et al., 2009; Vidal et al., 2009). In rodents, 5-HT4R activation shows antidepressant- and anxiolytic-like effects, and inhibition blocks fluoxetine's effect (Lucas et al., 2007; Mendez-David et al., 2014b). In unmedicated patients with MDD, we demonstrated that cerebral 5-HT4R binding is 6–8% lower than in healthy controls (Köhler-Forsberg et al., 2023).

We know that 5-HT4R levels in the neocortex and hippocampus are 10–12% higher in healthy men than in women (Clausen et al., 2024). Women might be affected by exogenous sex hormone exposure, e.g., oral contraceptives (Clausen et al., 2024; Larsen et al., 2020). Also, our previous observations in healthy men support that plasma testosterone is negatively associated with global brain 5-HT4R levels, and plasma estradiol is positively associated with amygdala 5-HT4R levels (Perfalk et al., 2017).

Here, we evaluate whether estradiol and/or testosterone levels in unmedicated men with MDD are associated with cerebral 5-HT4R levels, and if this association differs from that in healthy men.

## 2 Methods

### 2.1 Populations

We included data from 25 males with unmedicated moderate to severe depression and 98 healthy males aged 18–50. The data from depressed men came from the NeuroPharm study (Köhler-Forsberg et al., 2020), an open-label clinical trial (NCT02869035). A psychiatrist confirmed the MDD diagnosis based on ICD-10 criteria, and depression severity was assessed with the Hamilton Depression Rating Scale 17-item (HAMD_17_). Data from healthy men came from the Center for Integrated Molecular Brain Imaging (Cimbi) database at Copenhagen University Hospital, Rigshospitalet (Knudsen et al., 2016). All projects were approved by Ethics Committees (H15017713, H-1-2010-085, H-15017713, H-4-2011-103, H-6-2014-057, KF-01-2006-20, KF-21971/22025, KF-23830, KF-01-274821, KF-11-061/03, KF-01-124/04, KF-11-283038 and H-18038325).

### 2.2 Sex hormones

Plasma sex hormones were measured on the day of the positron emission tomography (PET) using a routine hospital laboratory at Rigshospitalet, Copenhagen University Hospital, from May 2006 to January 2019. Until November 2015 (26 healthy male PET participants), estradiol and testosterone were analysed using Cobas Estradiol II kits on a modular E170 and Cobas Testosterone-II kits on a Cobas e602 (Roche Diagnostic). Afterwards, the antibodies used for both analyses were changed to Elecsys® Estradiol-III on Cobas 8000 e602 and Elecsys® Cobas 8000, e801 (Roche Diagnostic). The new measurements were compatible with the previous by a conversion factor (validation reports can be obtained upon request). The new estradiol method resulted in a higher lower limit of quantification (LOQ) of 0.09 nM compared to 0.04 nM.

### 2.3 PET-imaging

The 25 men with MDD and 46 of the healthy men were scanned on the High-Resolution Research Topography (HRRT) Siemens PET-scanner (CTI/Siemens, USA) (256 × 256 × 207 voxels; 1.22 × 1.22 × 1.22 mm). Six healthy men were scanned on the 18-ring GE-Advance (General Electric, USA) with an approximate 6 mm in-plane resolution. Thirty-eight healthy subjects are from the previous study on the association between sex hormones and 5-HT4R levels in healthy men (Perfalk et al., 2017). Detailed procedure descriptions can be found (Marner et al., 2009). Briefly: PET-scans were obtained from a 120-min-dynamic acquisition immediately after a 20-s intravenous bolus injection of the [^11^C]SB207145 tracer below the mass dose limit of 4.5μg/70 kg bodyweight (Madsen et al., 2011). AIR 5.2.5 was used for motion correction (Woods et al., 1992).

High-resolution 3D T1-weighted magnetic resonance imaging was acquired on 2 S Magnetom 3-T scanners. It was segmented into the cerebrospinal fluid, white matter, and grey matter and co-registered to their corresponding PET images using SPM5 or SPM8 (Wellcome Centre for Human Neuroimaging, UCL, London, UK).

Regions of interest were automatically delineated by a user-independent algorithm in Pvelab (Svarer et al., 2005), and regional non-displaceable binding potentials (BP_ND_) were quantified by kinetic modelling of the mean tissue activity with the cerebellum (excluding vermis) as the reference region (Marner et al., 2009).

### 2.4 Statistics

We compared testosterone levels in the PET-scanned men with MDD and healthy controls using Welch's *t*-test, and estradiol was compared using Gehan's test due to the LOQ. Because half of the healthy PET participants had their estradiol measured with a different assay (see above), we, as a sensitivity analysis, compared the men with MDD to available measurements from healthy controls measured with the same assay (26 with PET, 47 without).

#### 2.4.1 Cerebral 5-HT4R BP_ND_ in a latent variable model

**Fig. 1:**
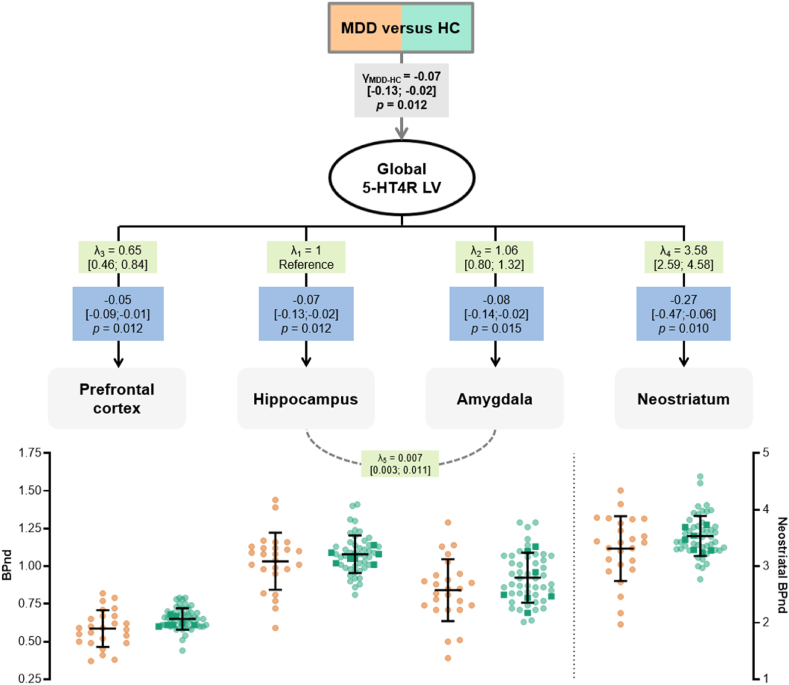
**Difference in cerebral 5-HT4R binding between men with MDD and healthy men** The estimated LVM shows the difference in cerebral 5-HT4R BP_ND_ between men with MDD and healthy controls (HC), which is tested through a global latent variable (global 5-HT4R LV). The difference in global 5-HT4R BP_ND_ between the groups is represented by γ and the regional loadings by λ. The blue boxes show individual regional differences with 95% CI in 5-HT4R BP_ND_ in men with MDD compared to healthy men. Initial diagnostic tests indicated a missing association between the hippocampus and amygdala (*p* = 0.001), which was added to the model structure of the LVM (λ_5_), which increased the modelled correlation between BP_ND_ hippocampus and amygdala. The regional 5-HT4R BP_ND_ (adjusted for age, injected tracer mass per kg bodyweight, and PET-scanner) are shown below with mean and SD (dark squares are HC subjects measured on the GE scanner).

We investigated 5-HT4R in the prefrontal cortex, hippocampus, amygdala and neostriatum due to their involvement in depression pathophysiology; their sensitivity to hormonal changes, expression of sex hormone receptors and aromatase (Barth et al., 2015; Azcoitia et al., 2021) and their density of 5-HT4R (Beliveau et al., 2017). We used a linear latent variable model (LVM, Fig. 1) framework to evaluate if the average global 5-HT4R BP_ND_ differs in men with MDD compared to healthy men. The cerebral 5-HT4R BP_ND_ was identified using a latent variable (LV) capturing the shared correlation in 5-HT4R binding between brain regions with high (neostriatum), intermediate (hippocampus and amygdala), and low binding (prefrontal cortex). The regional binding levels were independently adjusted for age, SB-injected mass/kg, and PET-scanner.

#### 2.4.2 Cerebral 5-HT4R BP_ND_ and sex hormones in a latent variable model

**Fig. 2:**
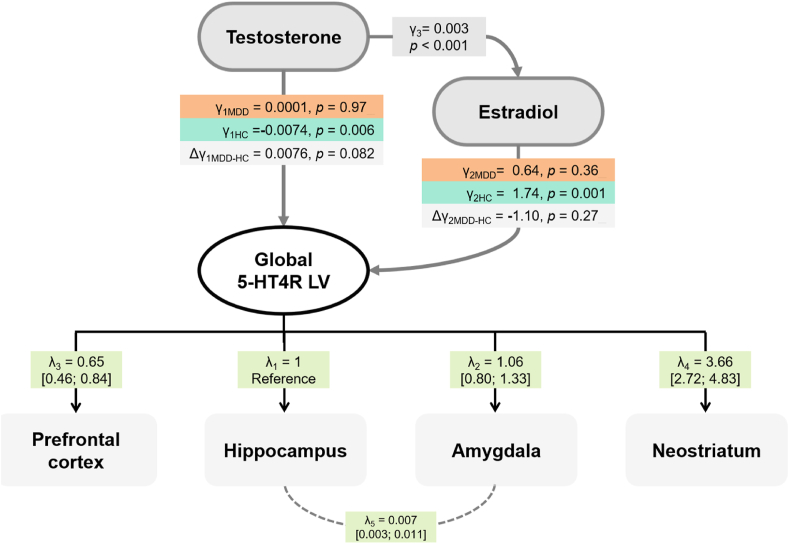
**The relationship between sex hormone levels and cerebral 5-HT4R binding in men with MDD and how it differs from healthy men** The estimated LVM where the effects of testosterone and estradiol on cerebral 5-HT4R BP_ND_ are tested through a global latent variable (global 5-HT4R LV). γ_1_ represents the testosterone effect, and γ_2_ the estradiol effect on the estradiol on the global 5-HT4R LV, where γ_3_ represents the effect of testosterone on estradiol levels. The Δγ represents the difference in the effects from men with depression (MDD) and in healthy men (HC). The corresponding regional loadings are represented by λ.

We then investigated the effect of testosterone on 5-HT4R BP_ND_ in men with MDD and whether it was mediated via estradiol, as estradiol in men is mainly derived from the aromatisation of testosterone (Vermeulen et al., 2002). We fitted a second LVM (Fig. 2) with an added effect of testosterone on estradiol and the 5-HT4R LV, as well as an effect of estradiol on the 5-HT4R LV to the previous LVM. The relation between estradiol and testosterone was assumed to be the same in MDD and healthy controls (Wit et al., 2021). However, the effect of testosterone and estradiol on the 5-HT4R LV could differ between MDD and healthy men. This was achieved by including estradiol (main effect) and the product between estradiol and MDD status (interaction effect) in the LVM. The estimated model parameters were used to deduce the direct effect of testosterone on the 5-HT4R LV and the indirect effect (mediated by estradiol), whose sum led to the total effect. The estimated effects in healthy men were compared to those in men with MDD. The estradiol LOQ was handled by assuming normally distributed concentrations and integrating the corresponding likelihood over possible estradiol values below the LOQ.

We checked QQ-plots for normality and score tests for misspecification in the residual covariance structure of the LVM (Holst and Budtz-Jørgensen, 2013). A covariance parameter corresponding to the misspecification with the smallest *p*-value was added to the model if it reached statistical significance after adjustment for multiple comparisons using Holm–Bonferroni, which was repeated until no association reached significance.

Six healthy men were scanned on the GE scanner, which was accounted for by including a scanner-covariate in our models. Because this could introduce a bias since no men with MDD were scanned on the GE scanner, we conducted sensitivity analyses restricted to subjects scanned on the HRRT (Supplementary Figs. 1–2).

For illustration purposes, we plotted group-stratified regional BP_ND_ and partial correlations with plasma testosterone adjusted for age, injected tracer mass per kg bodyweight, and PET-scanner in Supplementary Fig. 3.

#### 2.4.3 Post hoc network analysis of symptoms, sex hormones and 5-HT4R

To explore the possible reason for the uncoupling of 5-HT4R and sex hormones in depression med, we conducted a Gaussian Graphical Model with graphical LASSO regularisation (Briganti et al., 2024) (tuning = 0.5) based network analysis of 5-HT4R, sex hormones and core depressive symptoms (HAMD6 subscale) (Timmerby et al., 2017) and vegetative symptoms from HAMD (gastrointestinal symptoms, weight loss, and delayed, middle and initial insomnia) (Jensen et al., 2024a). We selected neostriatal 5-HT4R BP_ND_ (adjusted for age and tracer dose) as this region had the highest variance and loading (λ) in the LVM. Testosterone and estradiol (as a binary variable: above and below LOQ) were adjusted for age. We included all the men with MDD (n = 26; the previously excluded subject with high tracer mass dose only contributes data on sex hormones and symptom scores in this model). Centrality measures (betweenness and strength) were calculated to identify the most influential nodes in the network.

We present two-sided 95% confidence intervals (CI) and *p*-values unadjusted for multiple comparisons with *p*-values<0.05 considered statistically significant. Statistics were done in *R* 4.3.2 [R Core Team 43] with *Lava* to model the LVM (Holst and Budtz-Jørgensen, 2013) and *BuyseTest* for Gehan's test (Ozenne and Peron, 2022).

## 3 Results

The demographics, psychometrics, and radiotracer data in the males with MDD and healthy controls are in Table 1. Patients had a 37% lower injected tracer dose, which was adjusted for in subsequent analyses. More men with MDD had estradiol below the LOQ (i.e., ≤0.09 nM; 56%) than the PET-scanned healthy men (8%, *p* = 0.044). The proportions were very similar (56% vs 51%) when compared to all healthy men with the same estradiol measurement method instead of restricted to those with a PET scan. Testosterone was similar between men with MDD and healthy men.

**Table 1:** **Demographic and clinical profile** Data presented as mean (standard deviation) or *n* (%).

	**Men with MDD**	**Healthy**	**controls**
	(n = 25)	PET only (n = 52)	Hormone only (n = 73)
**Demographic**			
Age (years)	27.2 (7.9)	26.5 (5.9)	29.3 (6.8)
Body mass index (kg/m^2^)	24.4 (4.0)	23.5 (2.5)	a23.8 (2.6)

**Clinical**			
HAMD_17_	21.9 (3.2)		
Recurrent depression, *n* (%)	12 (48%)		

**Plasma hormone levels**			
Testosterone (nM)	16.7 (7.4)	17.9 (5.7)	16.5 (5.1)
Estradiol >0.09 nM (nM)	0.13 (0.02)	0.11 (0.03)	0.13 (0.03)
Estradiol ≤0.09 nM, *n* (%)	14 (56%)	5 (8%)	38 (51%)

**PET radiotracer**			
Injected SB dose (μg/kg)	0.012 (0.011)	0.019 (0.015)	

a BMI was only available for 65.

### 3.1 Lower cerebral 5-HT4R BP_ND_ in men with MDD

As previously shown in the Neuropharm-study, i.e. a group of primarily women (72%) with unmedicated MDD (Köhler-Forsberg et al., 2023), lower cerebral 5-HT4R BP_ND_ was also observed when we only compared men with MDD to healthy men. The average group difference in global 5-HT4R LV was −0.07 (CI; −0.13; −0.02, *p* = 0.012) with lower regional bindings in men with MDD (Fig. 1).

Six healthy men were scanned on the GE scanner, which was accounted for by a scanner covariate. The results (−0.08, CI -0.14; −0.01, *p* = 0.017) were similar in a sensitivity analysis of only subjects scanned on the HRRT (Supplementary Fig. 1).

### 3.2 Associations between testosterone and cerebral 5-HT4R BP_ND_ differed between men with MDD and healthy men

Testosterone and estradiol were added to the LVM to examine the relationships between hormonal levels and global LV binding in men with MDD (Fig. 2). However, we experienced estimation issues when fitting the LVM. We simplified the LVM by only accounting for the LOQ of estradiol in the main estradiol effect but not in the interaction effect.

We found no statistically significant association between cerebral 5-HT4R BP_ND_ and testosterone (γ_1MDD_ = 0.0001, CI: −0.006; 0.007, *p* = 0.97) nor estradiol (γ_2MDD_ = 0.64, CI: −0.75; 2.03, *p* = 0.36) in men with MDD. The association between estradiol and testosterone and cerebral 5-HT4R binding in healthy men (Fig. 2) largely resembles the reported in Perfalk et al., 2017) as the populations overlap (Perfalk et al., 2017). As Perfalk et al., we found a direct negative effect of testosterone on the global LV (γ_1HC_ = −0.0074, CI: −0.013; −0.002, *p* = 0.006). In addition, we found evidence of a positive association between estradiol and the global LV (γ_2HC_ = 1.74, CI: 0.71; 2.76, *p* = 0.001). Intriguingly, these associations are in opposite directions, which may explain the attenuated total testosterone effect on the global LV (γ_4HC_ = −0.0074 + 1.74∗0.003 = 0.002, CI: −0.008; 0.003, *p* = 0.40).

There was a trend towards a difference in the direct effect of testosterone (i.e. when disregarding the contribution from estradiol) on the global LV of 5-HT4R (γ_1MDD-HC_ = 0.0076, CI: −0.001; 0.016, *p* = 0.082) between men with MDD and healthy men (Fig. 2, group stratified partial correlations of regional 5-HT4R and testosterone levels are displayed in Supplementary Fig. 3). However, there were no statistically significant group differences in the associations between estradiol (γ_2MDD-HC_ = −1.10, CI: −3.04; 0.85, *p* = 0.27) or total testosterone (γ_4MDD-HC_ = 0.004, CI: −0.004; 0.013, *p* = 0.30).

The results were similar in a sensitivity analysis of only subjects scanned on the HRRT (Supplementary Fig. 2). In another sensitivity analysis, the LOQ in estradiol was accounted for in the main and interaction effects. However, no age adjustment was performed, nor was the covariance parameter from the score tests added. This led to similar estimates (relative difference between −22.5% and 22.4%) and *p*-values (difference ranging between −0.055 and + 0.020).

### 3.3 Sex hormones link vegetative symptoms to cerebral 5-HT4R BP_ND_ in men with MDD

In an exploratory post hoc network analysis of vegetative symptoms (gastrointestinal symptoms, weight loss and insomnia), sex hormones and neostriatal 5-HT4R BP_ND_ in depressed men, we observed positive associations between vegetative symptoms and testosterone, testosterone and estradiol, and estradiol and 5-HT4R (Supplementary Fig. 4). Core depressive symptoms (HAMD6) were not identified as part of the network. Testosterone and estradiol had high betweenness (1.1) and substantial strength (1.1 and 0.7, respectively) on centrality measures, supporting them as bridging variables between the vegetative symptoms and cerebral 5-HT4R levels.

## 4 Discussion

In addition to the previously shown negative association between global 5-HT4R BP_ND_ and testosterone in healthy males (Perfalk et al., 2017), we also observed a positive association with estradiol. As expected, from previously reported results in a mixed group of male and female patients with MDD (Köhler-Forsberg et al., 2023), the males with depression showed lower cerebral 5-HT4R than healthy males. We did not find evidence for an association between either testosterone or estradiol and cerebral 5-HT4R in men with MDD.

### 4.1 5-HT4R and sex hormones

Estradiol targets serotonergic neurons and can modulate the brain's serotonin system through multiple mechanisms at the gene expression level (Barth et al., 2015; Bethea et al., 2002). Specifically, estradiol increases 5-HT4R mRNA in anterior pituitary cell cultures (Papageorgiou and Denef, 2006) and cortical 5-HT2AR in rodents (Sumner and Fink, 1998; Fink et al., 1999; Sumner et al., 2007). Cortical 5-HT2AR also increases following estradiol treatment in postmenopausal women (Kugaya et al., 2003; Moses-Kolko et al., 2003). In line with this, we have previously shown that estradiol levels are positively associated with neocortical 5-HT2AR levels in healthy men (Frokjaer et al., 2010). We show that estradiol is positively associated with cerebral 5-HT4R in healthy men, not only in the amygdala, as previously observed (Perfalk et al., 2017). We do not observe an association between estradiol and 5-HT4R in men with MDD, although other studies suggest that estradiol may directly induce 5-HT4R gene expression (Papageorgiou and Denef, 2006).

In contrast to healthy men, we did not observe substantial evidence for a correlation between estradiol and testosterone with cerebral 5-HT4R binding in men with MDD. Although, possibly due to insufficient statistical power (the MDD group was about half the size of the healthy group), it could suggest that the hormonal association with cerebral 5-HT4R levels is reduced during depression in men. This may be related to decreased androgen receptors, as observed post-mortem in the hypothalamus of depressed patients (Wang et al., 2008), or lower brain aromatase activity, leading to lower local conversion of testosterone to estradiol. Postmortem findings of lower hypothalamic aromatase levels in men and women with MDD indirectly support the notion of altered aromatase activity (Wu et al., 2017) and aromatase gene polymorphisms being associated with depressive symptoms in women (Garcia‐Segura, 2008; Ancelin et al., 2020). We also speculate that the reason for the negative association in the healthy state can be due to the direct activation of androgen receptors, which are especially abundant in the amygdala, hippocampus, and neocortex (Giltay et al., 2012), leading to an inhibition of 5-HT4R gene expression.

Alternatively, the decoupling of an association between testosterone and 5-HT4R levels observed in men with depression may signify the presence of additional mechanisms within the pathophysiology of MDD. In our previous investigation of this same cohort, testosterone levels were positively associated with overall depression severity, but this association was primarily driven by vegetative symptoms rather than core depressive symptoms (Jensen et al., 2024a). In our exploratory network analysis, testosterone and estradiol were key bridges between vegetative symptoms and 5-HT4R, with strong network edges and high centrality. This suggests a disrupted *sex-hormone-serotonin homeostasis* during depression, where testosterone's effects on 5-HT4R are modulated by vegetative symptoms, potentially disrupting the typical neuroendocrine pathway observed in healthy men. This in consistent with the hypothesis that the metabolic consequences of melancholic depression disrupt or overshadow the more subtle link between testosterone and 5-HT4R present in healthy men. Further this may also reflect that testosterone's relationship with depression involves multiple pathways - both metabolic and neuroendocrine (Team RC, 2024).

### 4.2 5-HT4R and depression in men

Human in vivo imaging supports that serotonergic neurotransmission is affected in MDD. A study, primarily in men (∼84%), showed that synaptic serotonin release after the D-amphetamine challenge is lower in depressed patients than healthy controls (Erritzoe et al., 2023). Our group has shown that in a group of primarily women (∼28% men) with unmedicated MDD, the cerebral 5-HT4R level is 6–8% lower in patients than controls (Köhler-Forsberg et al., 2023). Here, we confirm the phenomenon in the male-only group.

Preclinical and clinical evidence suggests that 5-HT4R levels are sensitive to serotonergic manipulations in healthy controls such that higher synaptic serotonin, lower 5-HT4R (Haahr et al., 2014); however, this is not necessarily the case in depression and does not necessarily translate to absolute 5-HT4R levels in stable conditions. Indeed, changes in hippocampal 5-HT4R binding during SSRI treatment showed sex-specific patterns in relationship to structural hippocampal changes, suggesting sex-specific mechanisms in how the serotonin system adapts to intervention (Jensen et al., 2024b)**.**

### 4.3 Methodological considerations

These findings should be interpreted in the light of several limitations. Primarily the few male subjects with MDD, which limits power to capture small effects. Secondarily, we used total testosterone and estradiol levels. Only 1–3% of total testosterone and estradiol are unbound from sex hormone-binding globulin and albumin and are physiologically active, yet total levels correlate with their free hormone levels (Johnson et al., 2013; Mounib et al., 1988; Södergard et al., 1982). The men in this study were primarily younger (mean age 27); thus, our results might not generalise to older men or men in andropause.

### 4.4 Conclusions

We show that cerebral 5-HT4R levels are lower in men with MDD than in healthy men. In contrast to healthy men, we observed no discernible correlation between estradiol and testosterone with cerebral 5-HT4R binding in men with MDD, which suggests that the association between sex hormones and cerebral 5-HT4R levels is disrupted in the depressed state in men. This highlights a potential change in the coupling between sex hormones and the serotonergic system during depression, which we speculate may be due to the metabolic consequences of depression overshadowing the more subtle link between testosterone and 5-HT4R present in healthy men.

## Data availability

The data analysed in this study is subject to the following licenses/restrictions: A Cimbi database application is required to access the dataset through the following procedures: https://cimbi.dk/index.php/documents/category/3-cimbi-database.

## Author contributions

MRA, KRJ, and VGF contributed to the study's conception and design. MRA and KRJ wrote the initial manuscript draft and contributed equally to this paper. MRA, SVL, BO, and KRJ performed the statistical analyses. All authors contributed to the interpretation of the analyses and the manuscript revision and have approved the submitted version. Co-first authors can prioritise their names when adding this paper to their résumés.

## Funding

MRA was supported by the Independent Research Fund Denmark (DFF-1149-00007B), and SVL by the Innovation Fund Denmark (grant nos. 4108-00004B and 0134–00278B). KHRJ and VGF by the Research Fund of the 10.13039/100020584Mental Health Services - 10.13039/501100005275Capital Region of Denmark and The 10.13039/501100003554Lundbeck Foundation alliance BrainDrugs (R279-2018-1145).

## Declaration of competing interest

MBJ has given talks sponsored by H. Lundbeck and Boehringer Ingelheim. GMK has served as a consultant for SAGE Therapeutics and Sanos. VGF has given lectures for Lundbeck A/S, Jannsen-Cilag A/S, Gedeon Richter A/S, and Ferring Pharmaceuticals A/S. The other authors declare no competing interests.
