# Supplementary material for: Sex hormones and serotonin 4 receptor brain binding in men with and without major depressive disorder
Source: Neurosci Appl. 2025 Feb 28;4:105517. doi: 10.1016/j.nsa.2025.105517 (PMC12244137; doi:10.1016/j.nsa.2025.105517)

# Supplementary Materials

## Sup. Fig. 1: Difference in cerebral 5-HT4R binding between men with MDD and healthy men – a sensitivity analysis in men only imaged on the HRRT

The primary analysis presented in Figure 1 includes six healthy males who were imaged on the GE PET scanner, while the other 46 healthy males and all males with MDD were imaged on the more sensitive HRRT PET scanner. This was accounted for with scanner type as a covariate. However, this could introduce a bias. We, therefore, conduct a sensitivity analysis only on subjects imaged on the HRRT (HC=46, MDD=25). The regional 5-HT4R BP_ND_ were adjusted for age and injected tracer mass per kg bodyweight (not shown).


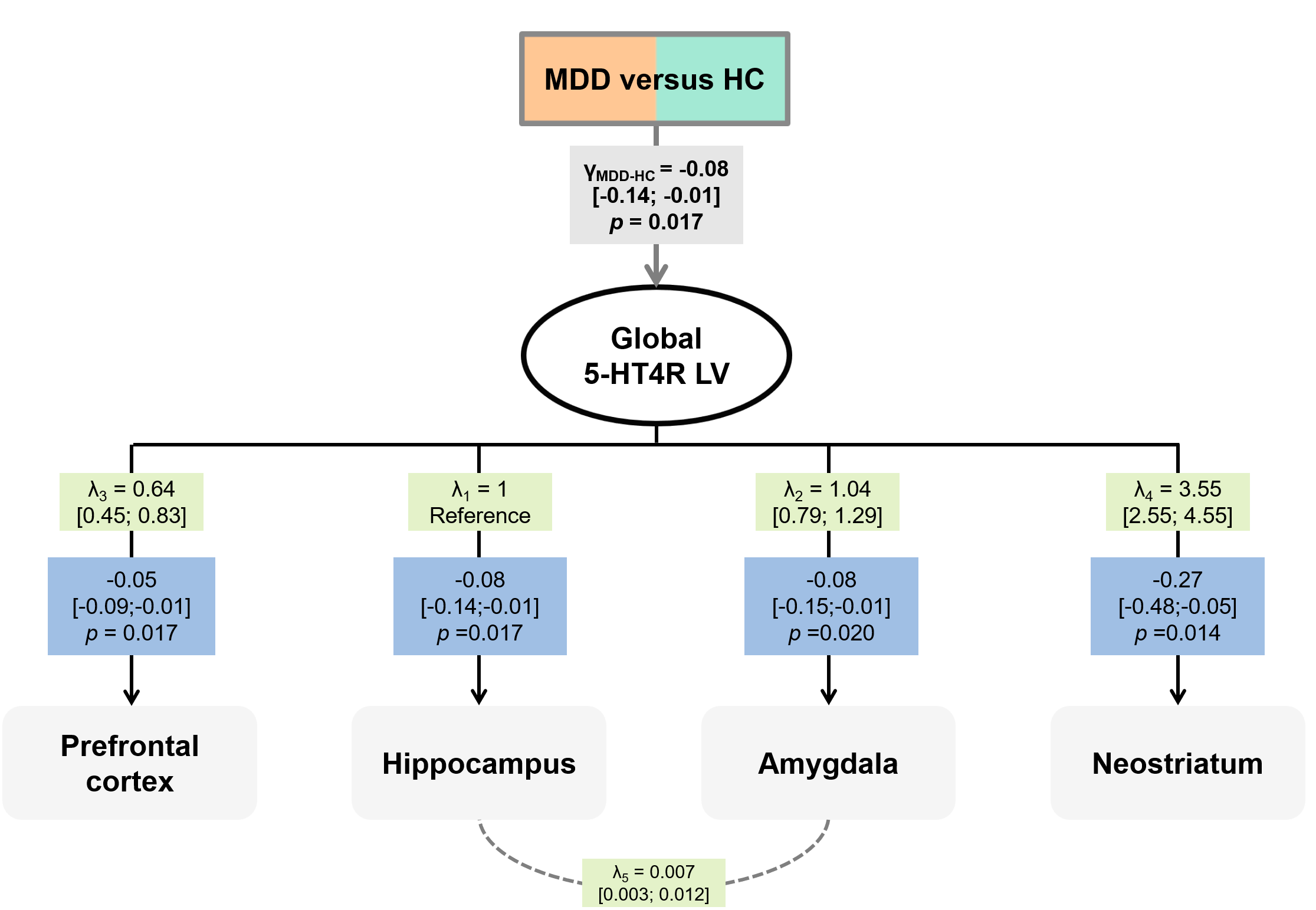


## Sup. Fig. 2: The relationship between sex hormone levels and cerebral 5-HT4R binding in men with MDD and how it differs from healthy men – a sensitivity analysis in men only imaged on the HRRT

The primary analysis presented in Figure 2 includes six healthy males who were imaged on the GE PET scanner, while the other 46 healthy men and all males with MDD were imaged on the more sensitive HRRT PET scanner. This was accounted for with scanner type as a covariate. However, this could introduce a bias. We, therefore, conduct a sensitivity analysis only on subjects imaged on the HRRT (HC=46, MDD=25). The estimated latent variable model where the effects of testosterone and estradiol on cerebral 5-HT4R BP_ND_ are tested through a global latent variable (global 5-HT4R LV). γ_1_ represents the testosterone effect and γ_2_ the estradiol effect on the estradiol on the global 5-HT4R LV, where γ_3_ represents the effect of testosterone on estradiol levels. The Δγ represents the difference in the effects from men with depression (MDD) and in healthy men (HC). The corresponding regional loadings are represented by λ.


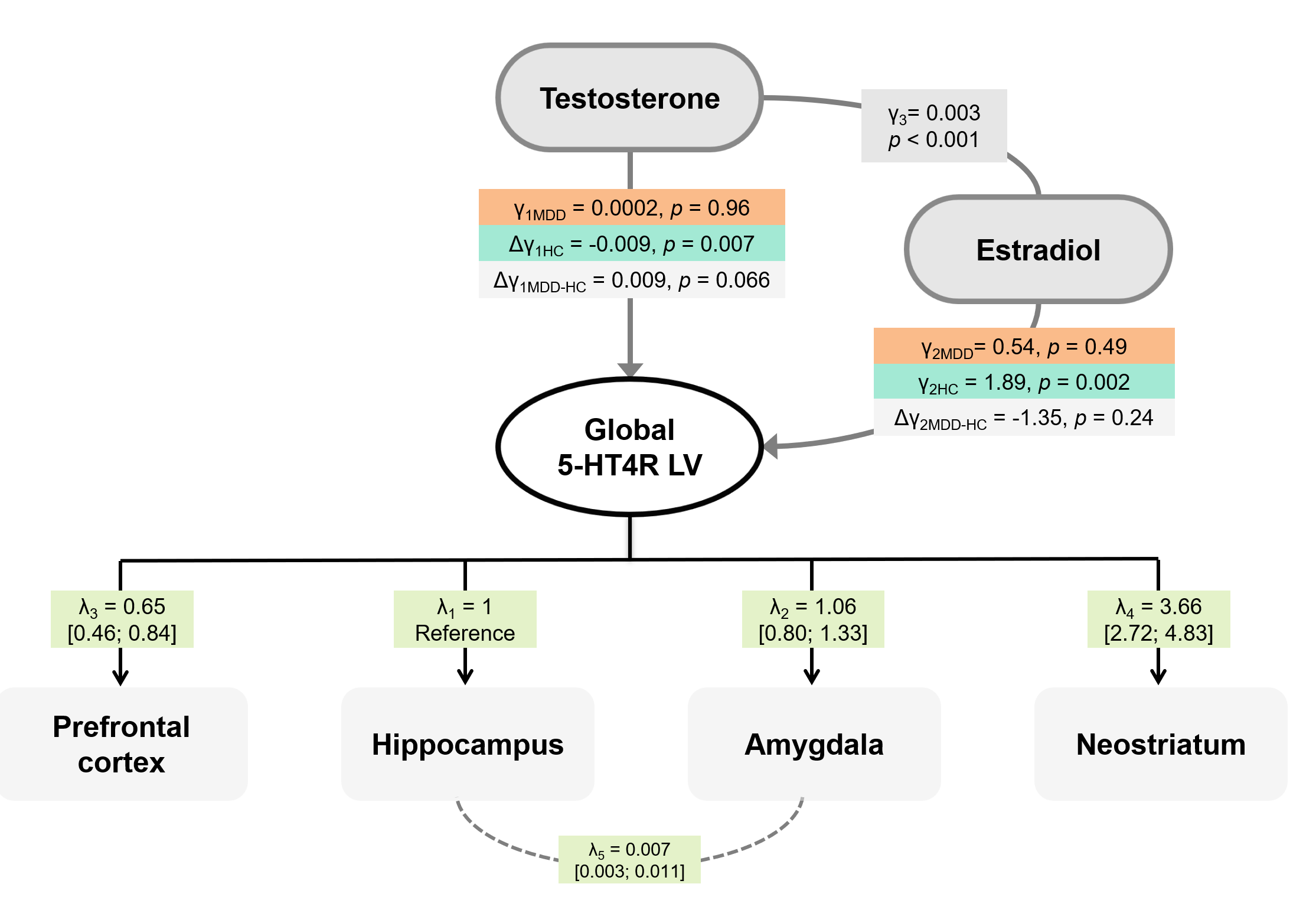


## Sup. Fig. 3: Regional 5-HT4R BP_ND_ and testosterone levels


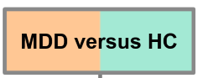
Plotted group-stratified regional BP_ND_ and partial correlations with plasma testosterone (nM) adjusted for age, injected tracer mass per kg bodyweight, and PET-scanner (dark squares are HC subjects measured on the GE scanner). These estimates are not from the LVM.


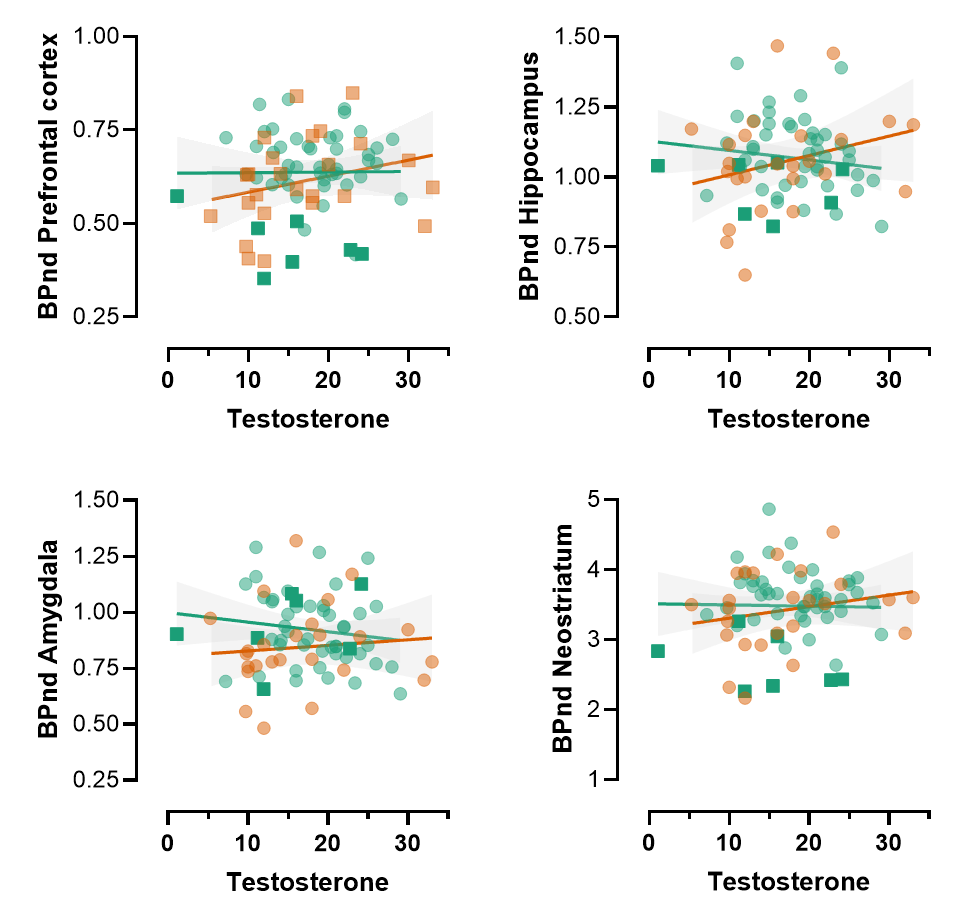


## Sup. Fig. 4: Exploratory network analysis of testosterone, neostriatal 5-HT4R BP_ND_, and symptoms in unmedicated men with MDD.

Network nodes represent testosterone and estradiol levels (adjusted for age), neostriatal 5-HT4R binding (adjusted for age and injected tracer mass), and Vegetative (gastrointestinal symptoms, weight loss, and delayed, middle and initial insomnia) and Core depressive symptoms. Edge strength is displayed above the lines, with thickness representing the strength of regularised partial correlations. Core depressive symptoms (HAMD6) were not identified as part of the network, and no other connections between network nodes were identified.

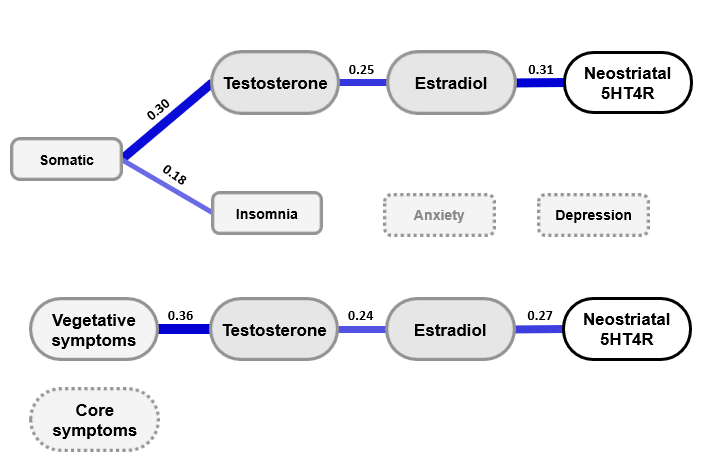

Supplement: Multimedia component 1 [file mmc1.docx]
